# Performance of biomarkers NF-L, NSE, Tau and GFAP in blood and cerebrospinal fluid in rat for the detection of nervous system injury

**DOI:** 10.3389/fnins.2023.1285359

**Published:** 2024-01-16

**Authors:** Katerina Vlasakova, Takayuki Tsuchiya, Ivy N. Garfinkel, Michael P. Ruth, Cheryl Tyszkiewicz, Theodore J. Detwiler, Christopher J. Somps, Lorenzo Di Cesare Mannelli, Warren E. Glaab

**Affiliations:** ^1^Merck & Co., Inc., Rahway, NJ, United States; ^2^Drug Safety Research and Development, Pfizer, Groton, CT, United States; ^3^Department of Neuroscience, Psychology, Drug Research and Child Health, University of Florence, Florence, Italy

**Keywords:** nervous system injury, CSF, biomarker, neurofilament light, glial fibrillary acidic protein, total Tau, neuron specific enolase, rat

## Abstract

**Background:**

Target organ toxicity is often a reason for attritions in nonclinical and clinical drug development. Leveraging emerging safety biomarkers in nonclinical studies provides an opportunity to monitor such toxicities early and efficiently, potentially translating to early clinical trials. As a part of the European Union’s Innovative Medicines Initiative (IMI), two projects have focused on evaluating safety biomarkers of nervous system (NS) toxicity: Translational Safety Biomarker Pipeline (TransBioLine) and Neurotoxicity De-Risking in Preclinical Drug Discovery (NeuroDeRisk).

**Methods:**

Performance of fluid-based NS injury biomarker candidates neurofilament light chain (NF-L), glial fibrillary acidic protein (GFAP), neuron specific enolase (NSE) and total Tau in plasma and cerebrospinal fluid (CSF) was evaluated in 15 rat *in vivo* studies. Model nervous system toxicants as well as other compounds were used to evaluate sensitivity and specificity. Histopathologic assessments of nervous tissues and behavioral observations were conducted to detect and characterize NS injuries. Receiver operator characteristic (ROC) curves were generated to compare the relative performance of the biomarkers in their ability to detect NS injury.

**Results:**

NF-L was the best performer in detecting both peripheral nervous system (PNS) and CNS injury in plasma, (AUC of 0.97–0.99; respectively). In CSF, Tau correlated the best with CNS (AUC 0.97), but not PNS injury. NSE and GFAP were suitable for monitoring CNS injury, but with lesser sensitivity. In summary, NF-L is a sensitive and specific biomarker in rats for detecting compound-induced central and peripheral NS injuries. While NF-L measurement alone cannot inform the site of the injury, addition of biomarkers like Tau and NSE and analysis in both blood and CSF can provide additional information about the origin of the NS injury.

**Conclusion:**

These results demonstrate the utility of emerging safety biomarkers of drug-induced NS injury in rats and provide additional supporting evidence for biomarker translation across species and potential use in clinical settings to monitor drug-induced NS injury in patients.

## 1 Introduction

Drug-induced nervous system injury (DINI) is often seen in both nonclinical and clinical studies with drug development candidates. Novel safety biomarkers can monitor such toxicities early and efficiently in nonclinical species and in some cases, may be translated to early clinical trials further ensuring patient safety. While accessible safety biomarkers for end-organ toxicities including liver, kidney or muscle have been evaluated across nonclinical species and in human, gaps remain in biomarkers for DINI. Recent availability of sensitive analytical assays presents an opportunity to evaluate and qualify novel biomarkers for the detection of DINI. The European Union’s Innovative Medicines Initiative (IMI) is a public-private partnership that has provided funds for two projects aimed at advancing research of nervous system (NS) biomarkers: Translational Safety Biomarker Pipeline (TransBioLine) and Neurotoxicity De-Risking in Preclinical Drug Discovery (NeuroDeRisk). The TransBioLine project has an overall goal of enabling implementation and regulatory acceptance of emerging fluid-based safety biomarkers for use in clinical trials and/or diagnosis of disease. The NeuroDeRisk project is aiming to provide novel integrated tools for improving the preclinical prediction of drug-induced adverse effects on the nervous system and help to de-risk drug candidates earlier in development.

As members of the TransBioLine NS and NeuroDeRisk peripheral nervous system (PNS) toxicity working groups, we have evaluated the performance of four translational biomarkers previously shown to be promising candidates for detection of neuronal diseases in patients: neurofilament light (NF-L), microtubule-associated protein tau (Tau), neuron specific enolase (NSE) and glial fibrillary acidic protein (GFAP). NF-L is an intermediate filament protein, part of the cytoskeleton, particularly abundant in axons and essential for the radial growth of axons, as well as maintenance of axon caliber and the transmission of electrical impulses along axons (Yuan et al., 2012). NF-L is emerging as a sensitive and specific biomarker in a wide variety of neurological diseases (Khalil et al., 2018). Tau is a neuronal microtubule-associated protein important for axonal transport. Tau and its phosphorylated forms are mainly studied for their role in Tauopathies (Holper et al., 2022). NSE is a cell specific isoenzyme of the glycolytic enzyme enolase, highly specific for neurons and peripheral neuroendocrine cells. NSE has been used as a biomarker for neuroblastoma and traumatic brain injury (TBI) (Isgro et al., 2015; Chen et al., 2022; Gardner et al., 2023). GFAP is an intermediate filament protein, unique to astrocytes, non-myelinating Schwann cells (PNS) and enteric glial cells and is mostly studied as a biomarker of TBI (Yang and Wang, 2015; Gardner et al., 2023).

The sensitivity of the biomarkers was evaluated in blood (plasma or serum) and cerebral spinal fluid (CSF) from rats in exploratory rat *in vivo* toxicity studies conducted with central nervous system (CNS) and PNS toxicants as well as with compounds inducing injury in organs other than NS. Histopathological as well as behavioral assessments were conducted to detect and characterize NS injuries. Such comprehensive characterization of biomarkers in a well-defined animal system can be used to compare and evaluate biomarker performance and provide useful information about biomarker sensitivity, specificity, and potentially the location of NS injury, especially in a field with no “gold standards” for detecting DINI. The four selected biomarkers are found both in rat and human and have sensitive, commercially available assays, therefore providing a unique opportunity to advance understanding of their performance and ultimately aid in their evaluation and regulatory acceptance for use in the clinic to ensure patient safety.

## 2 Materials and methods

### 2.1 *In vivo* rat studies

Studies were approved by the Merck & Co., Inc., Rahway, NJ, United States (MSD) or Pfizer Institutional Animal Care and Use Committee and conducted in an Association for Assessment and Accreditation of Laboratory Animal Care International–accredited facility in compliance with the National Institutes of Health (NIH) Guide for the Care and Use of Laboratory Animals. MSD studies were performed in Wistar-Han (Crl:WI[Han]) rats, and the Pfizer study with trimethytin (TMT) in Sprague–Dawley (Crl:CD[SD]) rats. The animals were acclimated, had access to water and food *ad libitum* and were randomized into treatment and control groups. The studies included necropsy time-points ranging from 6 h to 53 days and the study design often incorporated multiple necropsy time-points and doses. The compounds, route of administration, dosing frequency, vehicle, tissues histomorphologically assessed, dose levels, necropsy days, histopathologic outcomes and behavioral observations are presented in Table 1. Doses were calculated based on animal body weight, and the last dose was given to animals approximately 24 h prior to necropsy in studies with daily dosing. Animals were fasted overnight prior the necropsy and euthanized by exsanguination under isoflurane anesthesia (5 min induction at 5% isoflurane). Blood was collected via vena cava (MSD) or cardiac puncture (Pfizer). Blood and CSF samples were collected as terminal samples at necropsy and had a corresponding histopathologic examination. K_3_ EDTA plasma (14 MSD studies) or serum (one Pfizer study) and CSF samples were stored frozen at or less than −70°C until subsequent biomarker analyses. For simplification, the matrix in which the biomarkers were analyzed will be referred to as plasma throughout the manuscript.

**Table 1:** Study details and histopathological and observational findings.

Test article	Strain/dosing/frequency/vehicle	Tissues examined	Necropsy time (day)/dose in mg/kg	Histopathological findings (grade) [number of animals with finding/total number of animals per group]	Physical observations
*Studies with NS toxicity (sensitivity studies)*					
2-Chloropropionic acid (CPA)	Male Wistar, oral, single dose, water	B, NSc, NSa, DRG, SC, H, K, L, M, P, T	D2/750	No findings	Slow aerial righting reflex in 2/10 animals
			D3/750	Brain cerebellar necrosis (1–2) [7/19]	One animal early necropsy. Decreased activity, impaired or abnormal posture and gait, slow or impaired surface righting reflex convulsion-like activity, decreased temperature, no response to tail pinch rapid/labored breathing
MK-801or dizocilpine	Female Wistar, SC, single dose, 0.9% NaCl	B, NSc, NSa, DRG, H, K, L, M, P, T	D2/10	Brain neuronal necrosis (1) [12/12]	Convulsion-like activity, decreased activity, impaired righting reflex, recumbent, decreased skin turgor, rapid or labored breathing
Kainic Acid (KA)	Male Wistar, SC, single dose, 0.9% NaCl	B, NSc, NSa, DRG, K, L, M, P, T	D2/10	Brain neuronal necrosis (1–3) [4/12]	One animal early necropsy. Full body shakes (wet dog shakes after dosing) no abnormal observations before necropsy
Amphetamine (AMPH)	Male Wistar, SC, three doses day 1, 0.9% NaCl	B	D3/6.5	No findings	Behavior and gait signs on day 1, most normal on day 2, two found dead on day 3
			D3/10	No findings	Behavior and gait signs on day 1–3, 4/8 found dead on days 1–2
			D2/13	No findings	Behavior and gait signs on day 1 and 2, 3/8 found dead on days 1–2
Trimethyltin chloride*TMT	Male Sprague Dawley, IP, single dose day 1	B	D10/3	No findings	Lateral recumbency in 1/8
			D5-10/7	Brain (hippocampus) neuronal degeneration (1–4) [7/8]	Early necropsy due to physical signs in 5/8 animals, decreased activity, convulsions, hyperexcitability, aggression upon handling, ungroomed, changes in a locomotor activity assessment
2,5-Hexanedione (HXND)	Male Wistar, drinking water and oral, daily, water	B, H, K, L, M, T	D3/300	No findings	No observations
			D21/300	No findings	No observations
			D28/300	Testes seminiferous tubule degeneration (1) [3/8]	Dehydration
			D3/600	No findings	No observations
			D15-17/600	Testes seminiferous tubule degeneration (1) [12/16]	Early necropsy due to physical signs: dehydration, hypoactive, piloerection, splayed limbs, weight loss
3-Nitropropionic acid (NPA)	Male Wistar, IP, Twice daily, 0.9% NaCl	B, NSc, NSa, H, K, L, M, LI, SI, A	D8/2.5	Sciatic nerve degeneration (1) [1/4]	No observations
			D15/2.5	Sciatic nerve degeneration (1–3) [4/8] Saphenous nerve degeneration (1–3) [3/8]	No observations
			D8/7.5	Sciatic nerve degeneration (2–3) [3/4] Saphenous nerve degeneration (2–3) [3/4]	No observations
			D15/7.5	Sciatic nerve degeneration (1–3) [8/8] Saphenous nerve degeneration (1–3) [8/8]	Partial weight bearing and/or dragging of hindlimbs
			D5-8/12.5	Brain neuronal necrosis (1–3) [6/10] Sciatic nerve degeneration (1–4) [10/10] Saphenous nerve degeneration (2–4) [10/10]	2 Animals found dead; 5 early necropsy due to physical signs. No or partial weight bearing and/or dragging of hindlimbs, decreased activity, slowed respiration, recumbency
Acrylamide	Male Wistar, IP, 3 x weekly, 0.9% NaCl	B, NSc, NSa, DRG, SC, H, K, L, M, LI, SI	D23/40	Sciatic nerve degeneration (1) [3/6]	Decreased activity
			D37/40	Sciatic nerve degeneration (1) [5/6] Saphenous nerve degeneration (1) [4/6]	Decreased activity, ataxia of hindlimbs (1/6), decreased muscle tone (3/6), decreased number of lines crossed and rears, decreased forelimb grip strength
			D23/60	Sciatic nerve degeneration (1–2) [8/8]	Ataxia of hindlimbs (6/6), abnormal/ impaired gait (4/6), decreased muscle tone (3/6), decreased lines and decreased forelimb grip strength (−15%), increased hindlimb foot splay, decreased body
			D37/60	Sciatic nerve degeneration (2–3) [6/6] Saphenous nerve degeneration (1–2) [5/6]	Decreased activity (4/6), abnormal/impaired gait (6/6), decreased muscle tone (6/6), intermittent sternal recumbency (6/6), impaired righting reflex (5/6), distended abdomen (5/6), decrease in line crosses and rears, decreased forelimb grip, decreased body temp.
Doxorubicin	Male Wistar, IV, single dose, 0.9% NaCl	B, NSc, NSa, DRG, SC, H, K, L, M, P, T	D4/8	Testes seminiferous tubule degeneration (1–2) [7/8]	Decreased locomotor activity in 2–3/8, decreased hindlimb grip strength (5/8), small decrease in body temperature (5/8)
			D8/8	Testes seminiferous tubule degeneration (1) [8/8]	Decreased locomotor activity (7/8), decreased body temperature (8/8)
			D15/8	Sciatic nerve degeneration (1) [8/8] Saphenous nerve degeneration (1) [8/8] DRG degeneration (1) [8/8] Kidney tubule degeneration (1–3) [7/8] Testes seminiferous tubule degeneration (1) [8/8]	Decreased locomotor activity (5/8), piloerection (4/8), decreased muscle tone (6/8), small decrease in body temperature (4/8)
Cisplatin with 4%NaHCO_3_	Female Wistar, IP, once weekly 4 doses, 0.9% NaCl	B, NSc, NSa, DRG, H, K, L, M, P	D25/5	Kidney: tubule degeneration (1–3) [5/8]	5/8 Animals increased sensitivity to cold (cold water tail flick test)
			D53/5	No findings	No observations
*Studies with non-NS toxicity (specificity studies)*					
Merck A	Male Wistar, oral, daily, single, 0.5% MC	B, NSc, NSa, H, K, L, M, P, T	6 h/10	No findings	
			D2/10	Pancreas: acinus degeneration focal (1) [1/4]	No observations
			D4/10	No findings	No observations
			D18/10	No findings	No observations
			6 h/100	Pancreas: acinus degeneration focal (1–2) [4/4]	No observations
			D2/100	Pancreas: acinus degeneration focal (1) [4/4]	No observations
			D4/100	Pancreas: acinus degeneration focal (2) [4/4]	No observations
			D18/100	No findings	No observations
Puromycin	Male Wistar, IV, single, 0.9% NaCl	B, NSc, NSa, H, K, L, M, P, T	D4/150	No findings	Behavior and gait signs only immediately post injection in 3/8 animals
			D8/150	Kidney: tubule degeneration (2) [8/8], glomerulopathy (2) [8/8]	No observations
			D22/150	Kidney: glomerulopathy (2) [8/8]	No observations
Gentamicin	Male Wistar, IP, daily, 0.9% NaCl	B, NSc, NSa, H, K, L, M, P, T	D4/120	No findings	No observations
			D8/120	No findings	No observations
Methotrexate	Male Wistar, IP, once, PBS	B, NSc, NSa, H, K, L, M, P, T	D5/20	No findings	No observations
1-Cyano-2-hydroxy-3-butene (CHB)	Male Wistar, SC, once, 0.9% NaCl	B, NSc, NSa, H, K, L, M, P, T	D2/100	Pancreas: acinus single cell necrosis (2) [4/4]	No observations
			D3/100	Pancreas: acinus single cell necrosis (2) [3/4]	No observations
			D15/100	No findings	No observations

Route: IP, intraperitoneal; IV, intravenous; SC, subcutaneous. Vehicle: MC, methylcellulose in water; Tissues: B, brain; NSc, nerve sciatic; NSa, nerve saphenous; DRG, dorsal root ganglia; SC, spinal cord; H, heart; K, kidney; L, liver; M, muscle; P, pancreas; T, testes; SI, small intestines; LI, large intestines; A, aorta; *Study performed at Pfizer.

Necropsy was performed and select tissues were processed for histomorphologic examination. Tissue was fixed in 10% neutral buffered formalin, processed, and embedded in paraffin. Embedded tissues were cut into 4–6 micron sections and stained with hematoxylin and eosin. Stained tissue sections were examined microscopically, and severity grades were assigned using a score scale of 0 to 5: 0 (no observable pathology), 1 (minimal or very slight), 2 (mild or slight), 3 (moderate), 4 (marked), or 5 (severe).

A functional observational battery (FOB) was performed for all the sensitivity studies with the exception of the 2,5 hexanedione, amphetamine, and TMT studies. All studies incorporated post-dose and daily clinical animal observations. FOB typically consisted of home cage observations (activity level), hand-held observations (lacrimation, salivation, piloerection, palpebral closure, muscle tone), open field observations (number of line crosses, number of rears, posture, gait, convulsion, stereotypy) stimulus activity response (click response, approach response, touch response, tail pinch response, palpebral response, pinna response, pupillary response, surface and aerial righting reflex), forelimb or hindlimb grip strength measurement, body temperature and hindlimb foot splay. In the cisplatin study, a cold-water tail flick test was performed by submerging tails into water at approximately 4°C for up to 15 s. and measuring the withdrawal time.

### 2.2 Biomarker assays

Biomarkers were measured in plasma and CSF using Meso Scale Discovery (MSDIS) (Gaithersburg, MD) R-plex kits with the following exceptions: NF-L in CSF in the 2,5-hexanedione study was measured using the Quanterix (Billerica, MA) human NF-L assay. NF-L, Tau and GFAP were measured in serum and CSF in the TMT study using the Quanterix Neurology 4-plex at 1:4 dilution for serum and 1:40 for CSF. MSDIS R-plex kits were optimized for performance in rat plasma and CSF and fit-for-purpose validated to assess assay performance for parameters including intra and inter-assay precision, dilutional linearity, short and long-term stability, and calibrator performance. Biomarker cross-reactivity was confirmed on rat brain homogenates. NF-L was analyzed at a 1:2 dilution in plasma and 1:15 in CSF, using the human NF-L kit cat#F217X and MSDIS Diluent #12 as an assay diluent and Diluents #11 or #100 as an antibody diluent. NSE was analyzed at 1:40 dilution in plasma and 1:60 in CSF, using the human NSE kit cat#F212D and MSDIS Diluents #12/#11. GFAP was analyzed at 1:2 dilution in plasma and from a 1:5 to 1:15 dilution in CSF, using the human GFAP assay cat# F211M and MSDIS Diluents #101/#27. Tau was analyzed at a 1:2 dilution in plasma and from a 1:5 to 1:15 dilution in CSF, using the mouse total Tau assay cat# F228E and the MSDIS Diluents #11/#11 or Diluents #12/#12.

### 2.3 Statistical methods

For the comparison of biomarkers with different plasma concentrations and methods of detection, all measured values were converted to fold changes calculated from the average of each study control group. In cases where measured biomarker values were below the lower limit of quantification (LLOQ), such values were replaced by the lower limit of quantification value. Mean and 95% CI for each dose-group and time-point were calculated using GraphPad Prism 8.1.1 software. Receiver operating characteristic (ROC) curves were generated with GraphPad Prism 8.1.1 (Wilson/Brown method); using fold change values calculated from the average of controls of each study. ROC plots provide a statistical method to assess the diagnostic accuracy of a biomarker that has a continuous spectrum of test results. The ROC curve is a graphical display of the trade-offs of the true-positive rate (sensitivity) and false-positive rate (1 − specificity) corresponding to all possible binary tests that can be formed from this continuous biomarker. Each classification rule, or cut-off level, generates a point on the graph. The closer the curve follows the left-hand border and the top-border of the ROC space, the more accurate the test (Soreide, 2009). The sensitivity was calculated at 95% specificity where possible. A total of 19 CSF samples were eliminated from the dataset of approximately 450 samples due to a suspected brain matter contamination of CSF occurring during collection. The TMT study was included in the ROC analysis even though NSE was not measured. 2,5-hexanedione was not included in the ROC curve analysis since PNS was not examined. In the studies treated with known brain toxicant and where only brain was histomorphologically assessed, the assumption was that PNS was without findings for the purpose of the ROC analysis.

## 3 Results

### 3.1 *In vivo* studies outcome and biomarker performance

To assess the performance of the candidate biomarkers, rat *in vivo* studies were conducted using compounds known to induce NS injury (defined as degeneration/necrosis). Individual study outcome and endpoints assessed are summarized below. Biomarker responses for all following studies are shown in Figures 1A,B. Detailed FOB results are provided in the Supplementary Tables S1–S6.

**Figure 1 (Continued):**
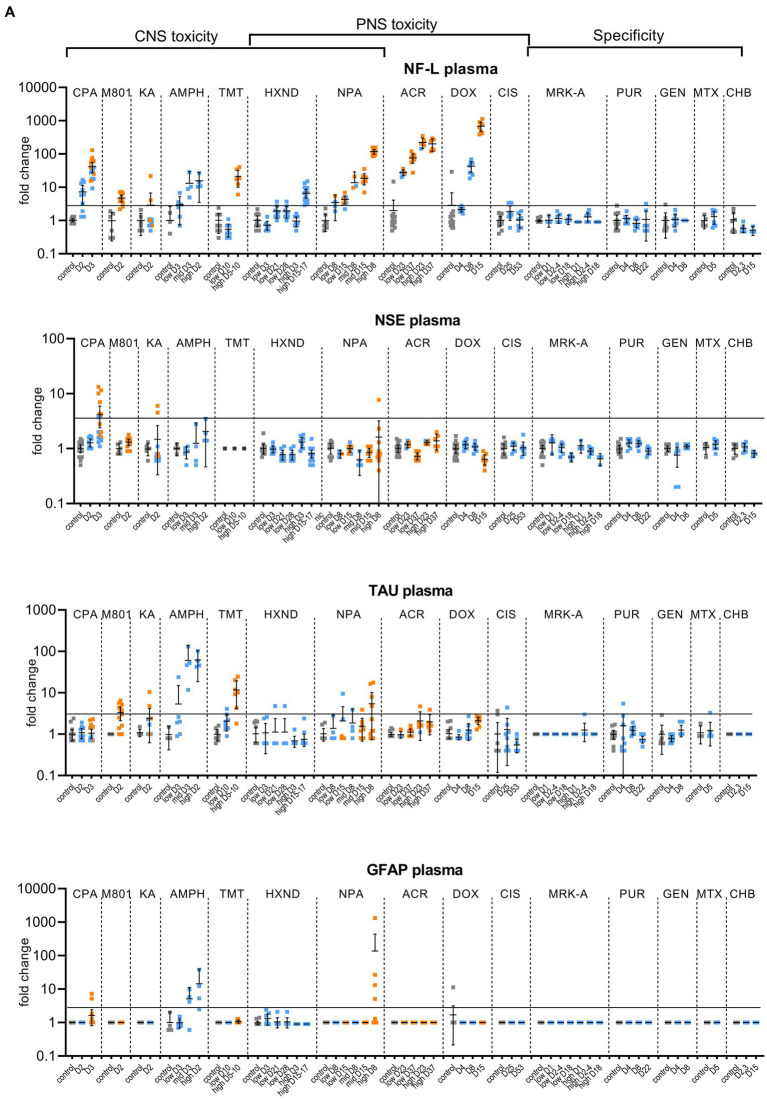


**Figure 1:**
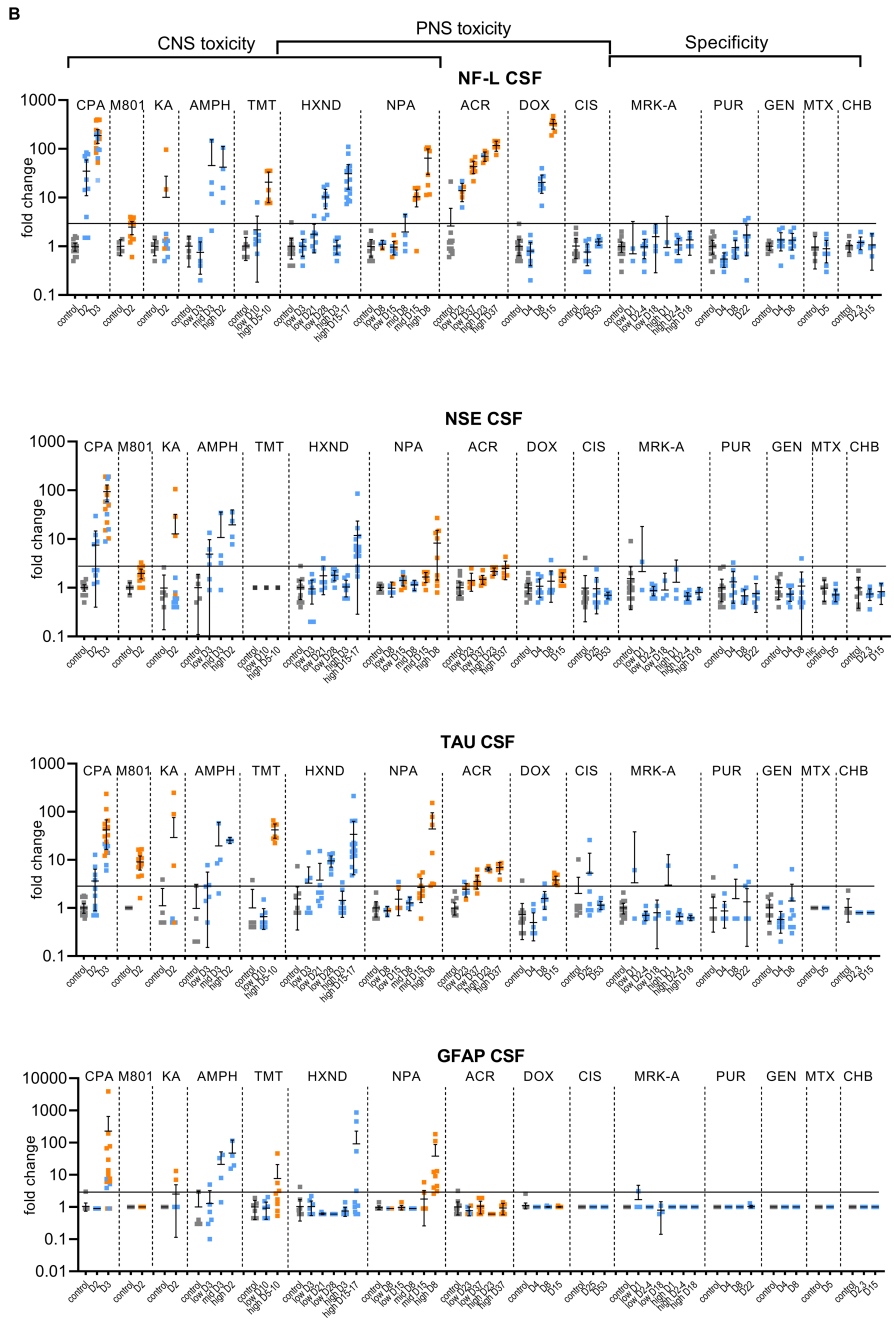
Biomarker performance across all sensitivity and specificity studies in plasma/serum **(A)** and in CSF **(B)**. Biomarkers changes are expressed as fold change to the average of each study control group and organized by study and study day or study dose as listed in Table 1. Study controls are in gray color, blue color signifies samples with no NS histopathological finding, orange color signifies histopathological finding of degeneration or necrosis of any severity in either CNS or PNS. The black line is placed at a 3-fold increase from controls, indicating possible cut-off values towards defining an injury in response to treatment. Data points that are placed exactly at 1-fold usually mean that the measured values were not quantifiable and therefore replaced by the lower quantification limit, resulting in no fold change.

#### 3.1.1 2-Chloropropionic acid (CPA)

No CPA-related clinical signs were noted on SD1 or SD2. On SD3, animals presented with decreased activity, abnormal gait, flattened/hunched posture, convulsion-like activity, red nasal discharge, head tilt, partially closed eyes, and rapid/labored breathing. In the FOB assessments on SD3, the following signs were observed in more than third of the tested animals: decreased activity in open field, splayed hindlimbs, sternal recumbency, swaying side-to-side, unsteady gait, ataxia, tonic convulsions, impaired and slow surface righting, no tail pinch response, and decreased body temperature (mean decrease of 1.3°C) compared to controls. There were no histopathological changes noted in any of the examined organs on SD2. On SD3, there was minimal (6 animals) to mild (6 animals) cerebellar necrosis. Microscopic findings in the cerebellum had a non-uniform lobular distribution in which some cerebellar lobules were affected (typically nearest the ventral midline) while others were spared. Findings consisted mostly of necrosis of the inner granule cells, accompanied by edema in the granular cell layer, necrosis of a smaller percentage of Purkinje cells, and edema in the Purkinje cell layer. In addition, individual necrotic neurons were observed in pontine, habenular, and olivary nuclei in some rats. These results were consistent with previously published changes (Simpson et al., 1996). NF-L increases in plasma and CSF were observed at 24 h. post dose and preceded both behavioral changes as well as observable microscopic changes. In plasma, NF-L was increased in most animals on SD2 and in all animals by SD3. NSE and GFAP were increased in some animals on SD3 only. CPA elicited robust response in CSF in all measured biomarkers on SD3 (average fold increase >10-fold) and also on SD2 for NF-L, NSE and Tau in some animals. Tau was not increased in plasma at any point despite large increases detected in CSF.

#### 3.1.2 MK-801

Post dose clinical observations were included with the FOB assessment on SD1 and included circling (onset ~3–4 min), falling side to side (onset ~3–4 min), recumbency (onset ~7–30 min) and sternal recumbency and motionlessness with eyes wide open (onset ~1.5 to 2.5 h post dose). Additional signs observed were decreased skin turgor, salivation, rapid or labored breathing, and red eye discharge. Histopathological evaluations revealed minimal neuronal necrosis in all treated animals, affecting individual neurons, localized to the posterior cingulate/retrosplenial cortex of the postero-medial aspect of the cerebrum of the brain consistent with previously published findings (Bueno et al., 2003). Necrosis was characterized by isolated neurons with angular brightly eosinophilic cytoplasm and condensed basophilic nuclei. Additionally, there were histopathologic changes in the pancreas (depletion of zymogen granules of the acinar cells), liver (hepatocellular vacuolation) and heart (acute and focal minimal to mild degeneration consistent with impaired tissue perfusion) that were attributed to the poor physical condition of the animals. Tau in CSF was the most responsive biomarker after MK-801 treatment with an average fold increase of approximately 9-fold and an increase in 11 out of 12 animals with the grade 1 brain finding. NF-L in plasma was increased in most animals (10 out of 12 animals) with an average fold increase of approximately 5-fold. Interestingly, NF-L in CSF was slightly increased above 3-fold only in a small subset of the animals.

#### 3.1.3 Kainic acid

Kainic acid (KA) was administered as per the study design reported previously by Srivastava (Srivastava et al., 2020). Treated animals were active and alert at 0.75 to 1-h post dose, while most control animals were asleep. Observations on SD1 present in at least one third of the animals included: full body shakes (wet dog shakes), head trembling, twitching, or jerking and chewing. Prior to necropsy on SD2, animals mostly presented as normal. One animal was sacrificed early due to poor physical condition at 2.75 h post-dose. Minimal to moderate neuronal necrosis was observed in 4 out of 12 rats. This was most pronounced in the hippocampus and piriform lobe of the cortex but was present throughout the cerebrum. The change was not observed in the brain stem or cerebellum. The neuronal necrosis was characterized by focally extensive regions of neurons with small amounts of angular, brightly eosinophilic cytoplasm and condensed basophilic nuclei. The more severely affected rats had associated spongiosis of the neuropil. The injury caused by KA treatment was very heterogeneous. Tau and NSE in CSF were increased in the animals with grade 3 findings, while only two of the grade 3 animals had increased plasma NF-L, NSE and Tau (Figures 1A,B). The animal that was terminated early at 2.75 h post dose already presented with an 8-fold increase of Tau in CSF and 12-fold increase of NSE in CSF correlating with a grade 3 finding of neuronal degeneration.

#### 3.1.4 Amphetamine

Amphetamine (AMPH) study was designed as a tolerability study and the treatment was not tolerated well, resulting in unscheduled deaths: at the high-dose in three animals at SD1-3, at the mid-dose in 4 animals at SD1-2 and at the low dose in 2 animals on SD3. Clinical observations in all groups included abnormal gait (walking backwards), circling, decreased activity, excitability to stimuli, increased activity, licking, unsteady gait, vocalization upon handling, decreased skin turgor, hunched posture, red discharge from the penis, splayed hindlimbs, red discharge from the nose, salivation, slowed respiration, red fur discoloration of forepaws, red fur discoloration of the muzzle, and urine staining generally beginning on SD1. Body temperatures were collected prior to dosing and approximately every 30 min after the first dose until approximately 4 h following the last dose. Generally, when body temperatures reached ~41.4°C, animals were cooled until their temperature returned to ~40.0°C. Elevated temperatures were observed in 2, 5 and 8 animals in the low, mid and high dose, respectively. Amphetamine dosing did not result in observable histopathology, but all biomarkers were increased in the CSF at the high and mid doses, on average > 10-fold. The low dose resulted in a modest increase (< 10-fold) in NSE, Tau and GFAP for some animals. In plasma, NF-L, Tau and GFAP increased with time and dose with the largest response in Tau (all 3 dose groups displayed increases >10-fold).

#### 3.1.5 Trimethyltin

Trimethyltin (TMT) was administered per a previously reported study design (Imam et al., 2018). The high dose was not well tolerated and resulted in early euthanasia (SD5-8) of 5 out of 8 animals. Clinical observations were limited to the high dose (one low dose animal was observed with lateral recumbency immediately post-dose) and consisted of decreased activity, hyperexcitability, aggression upon handling and ungroomed appearance. Locomotor activity assessment performed on SD 8 using the Kinder Scientific Motor Monitor (Tyszkiewicz et al., 2023) revealed increases in horizontal movements at the high dose compared with vehicle controls. Microscopic findings were limited to assessment of the brain and consisted of neuronal degeneration and necrosis in subiculum, CA1, CA2, and/or CA3 regions in 7 out of 8 animals administered 7 mg/kg TMT. Neuronal degeneration was not observed at the 3 mg/kg dose. NF-L and Tau were increased in the high dose animals both in CSF and serum, GFAP was not increased in serum and increased in only 2 animals in the CSF (Figures 1A,B). NSE was not measured.

#### 3.1.6 2,5-Hexanedione (HXND)

In the low dose, dehydration and body weight decrease was observed in a third of the animals. In the high dose group, clinical observations that affected more than a third of the animals included dehydration, scant stool, chromorhinorrhea, piloerection, and hypoactivity. PNS was not examined, however, HXND is known to induce central-peripheral axonopathies characterized by distal axon swelling and degeneration. Physical signs in some of the rats sacrificed early (splayed limbs, ataxia, and paralysis) were in agreement with observations attributed to NS findings in published studies (Spencer and Schaumburg, 1975; Couri and Milks, 1982). In our study, histopathologic findings were limited to the high dose, at SD15-17, and consisted of minimal vacuolation and minimal degeneration in seminiferous tubules in testes of 12 out of 16 animals as well as minimal sperm decrease, cellular debris in the lumen of the epididymides and hyaline droplets in the kidney tubules. There were no microscopic findings in the brain, but there was a robust biomarker response in the CSF that increased with time and dose. NF-L and Tau were increased in all samples from the early necropsy high dose group (SD15-17) and at SD28 in the low dose group. NSE in CSF was also increased in the majority of the high dose group animals (average fold-change 12-fold). In plasma, only NF-L was increased in the high dose (average fold change 7-fold) on SD15-17.

#### 3.1.7 3-Nitroproprionic acid (NPA)

There were no clinical signs at the low dose. In the mid-dose, animals displayed partial weight bearing and/or dragging of hindlimbs beginning on SD7, and in the high dose beginning on SD3. Individual animals with decreased activity, unkempt appearance, decreased skin turgor, slowed respiration, recumbency, and skin discoloration were observed beginning on SD5. FOB observations in the high dose were as follows: decreased muscle tone, hunched posture, splayed hindlimbs, ataxia, slow surface and aerial righting reflex, decreased body temperature and hindlimb grip strength. Test article-related histopathologic findings were present in the brain, sciatic nerve, saphenous nerve, cecum, and at the intraperitoneal injection site. In the brain, minimal to moderate neuronal necrosis was present in 6 out of 10 animals from the 12.5 mg/kg b.i.d. dose group. The neuronal necrosis was observed in the striatum (caudate putamen) and/or hippocampus in most animals as reported previously (Vis et al., 1999). A focal area in the cerebellum or thalamus was also affected in some animals. There was no evidence of brain neuronal necrosis in animals from the 7.5 or 2.5 mg/kg b.i.d. dose groups at either SD8 or SD15 necropsies. At SD5-8 (high dose) and SD8 (mid-dose), minimal to moderate axonal degeneration was present in both sciatic and saphenous nerves in most animals. Minimal axonal degeneration in the sciatic nerve was present in one animal in the low dose at SD8. At SD15, minimal to moderate axonal degeneration was present in both the sciatic and saphenous nerves at comparable severity in all animals in the mid dose group, and in a subset of animals in the low dose group.

NPA dosing resulted both in CNS and PNS injury; PNS injury was observed sooner and in lower doses than CNS injury. NF-L in plasma increased with time and dose appearing as early as SD8 in the low dose, even in individual animals without a corresponding histopathologic finding. GFAP and Tau increased only at the highest dose level in a subset of animals on SD5-8, the final timepoint measured for that group. Increases in the CSF were seen in all biomarkers (average fold change >10-fold), predominantly in the high dose where brain injury was present.

#### 3.1.8 Acrylamide (ACR)

Clinical signs observed in the low dose were limited to splayed hindlimbs and abnormal gait beginning on SD26. In the high dose, additional clinical signs were seen, including distended abdomen, hindlimb dragging, fecal staining, decreased skin turgor, splayed forelimbs, gasping, and audible respiratory sounds beginning on SD15. FOB observations in the low dose group prior to necropsy were decreased number line crosses and rears, decreased muscle tone and impaired aerial righting reflex. The same FOB observations were noted in the high dose with more severity and frequency. Histopathologic findings consisted of minimal to moderate axonal degeneration of peripheral nerves with the sciatic nerve being the most sensitive to acrylamide-related degeneration, followed by the saphenous nerve and caudal plexus. In general, the incidence and/or severity of the change progressively increased with dose and duration of treatment. No histopathologic changes were noted in the brain. The injury to PNS was best detected by NF-L with time and dose dependent increases both in plasma and CSF, reaching large fold changes (over 100-fold in plasma in high-dose) and with increases in individual animals in the low-dose without corresponding histopathologic findings. Tau increased in the CSF in a dose-dependent manner with 7-fold changes in the high doses at SD23 and SD37 and 4-fold in the low-dose for SD37. Remaining biomarkers were not increased.

#### 3.1.9 Doxorubicin (DOX)

Clinical signs were noted starting about SD9 and consisted of unkempt general appearance, salivation, and fecal staining. In the FOB, there were slight decreases in line crosses, rears and hindlimb grip in some animals at SD4. At SD7, the same findings continued with higher incidence. On SD14, there was still slight decreased mean locomotor activity, but this decrease was smaller than at SD7. Additional signs at SD14 were attributed to the glomerular toxicity and consisted of dried, black lacrimation, piloerection, and decreased muscle tone. There were no histopathologic findings in NS at SD4 and SD8. At SD15, very slight axonal degeneration was noted in the sciatic nerve of all animals and in the saphenous nerve in seven out of eight animals. In addition, very slight degeneration of the dorsal root ganglia (DRG) was observed in all animals, in agreement with previous reports (Jortner, 1988). Doxorubicin induced albuminuria beginning SD8 with TEM confirmed glomerulopathy and very slight to moderate kidney tubular degeneration in 7 out of 8 animals at SD15. Animals at all necropsy points also presented with predominantly minimal seminiferous tubule degeneration. Doxorubicin was the only study with documented DRG degeneration in addition to the nerve degeneration. NF-L increases reached the highest fold changes of all studies, up to 1,000-fold in plasma and 400-fold in CSF. NF-L in plasma and CSF was increased at SD8, preceding the NS histopathologic observation at SD15. Except for Tau showing an increase of approximately 4-fold at the high dose in CSF (SD15), no other biomarker was increased.

#### 3.1.10 Cisplatin (CIS)

There were no test article-related clinical signs. The FOB included a cold-water tail flick test to assess sensitivity to cold. When compared to the controls, an increase in tail sensitivity to cold water was noted in 5 out of 8 test-article treated animals at SD24. There were no other neurobehavioral findings observed. In the recovery group (SD52), there were no changes in the cold-water tail flick test or any other findings. Microscopically, no changes were observed in brain, dorsal root ganglia or nerves. In spite of sodium bicarbonate administrated to protect the kidneys from the known toxic effect of cisplatin, (Guindon and Hohmann, 2013), minimal to moderate renal tubular degeneration was present at necropsy at SD25 but not at the recovery necropsy. The cisplatin study was designed to induce peripheral neuropathy. There were no behavioral changes observed except for increased sensitivity to cold, nor histopathological degenerative changes in the NS. None of the evaluated biomarkers correlated with the finding of cold sensitivity. Tau in CSF was variable, but increases were not consistent or time/dose dependent.

#### 3.1.11 Specificity studies

Four specificity studies were conducted with compounds targeting non-NS organs with microscopic evaluation of brain and saphenous and sciatic nerves in addition to additional tissues (listed in Table 1). The MRK-A study induced very slight to slight pancreatic acinar cell toxicity at necropsy at 6 h, SD2 and SD3. The puromycin treatment resulted in slight glomerulopathy on SD8 and SD22 and kidney tubular degeneration in all animals at SD8. The gentamicin study did not present with any degenerative postmortem changes in any of the examined tissues. A methotrexate study was designed to induce slight liver toxicity. Even though methotrexate can cause CNS toxicity in rats (Berlin et al., 2020), the dose used was too low to cause CNS injury. Histopathological assessment showed no changes and no physical signs; therefore, the methotrexate study was categorized as a specificity study. The acute pancreatic toxicant 1-cyano-2-hydroxy-3-butene caused pancreatic acinar cell toxicity grade 3 and 4 at SD2 and SD3.

None of the analyzed biomarkers were consistently increased in these studies, even though there was some variability in individual animals, mostly seen in the CSF (Figures 1A,B).

### 3.2 Receiver operating characteristics curve and correlation analyses

**Figure 2:**
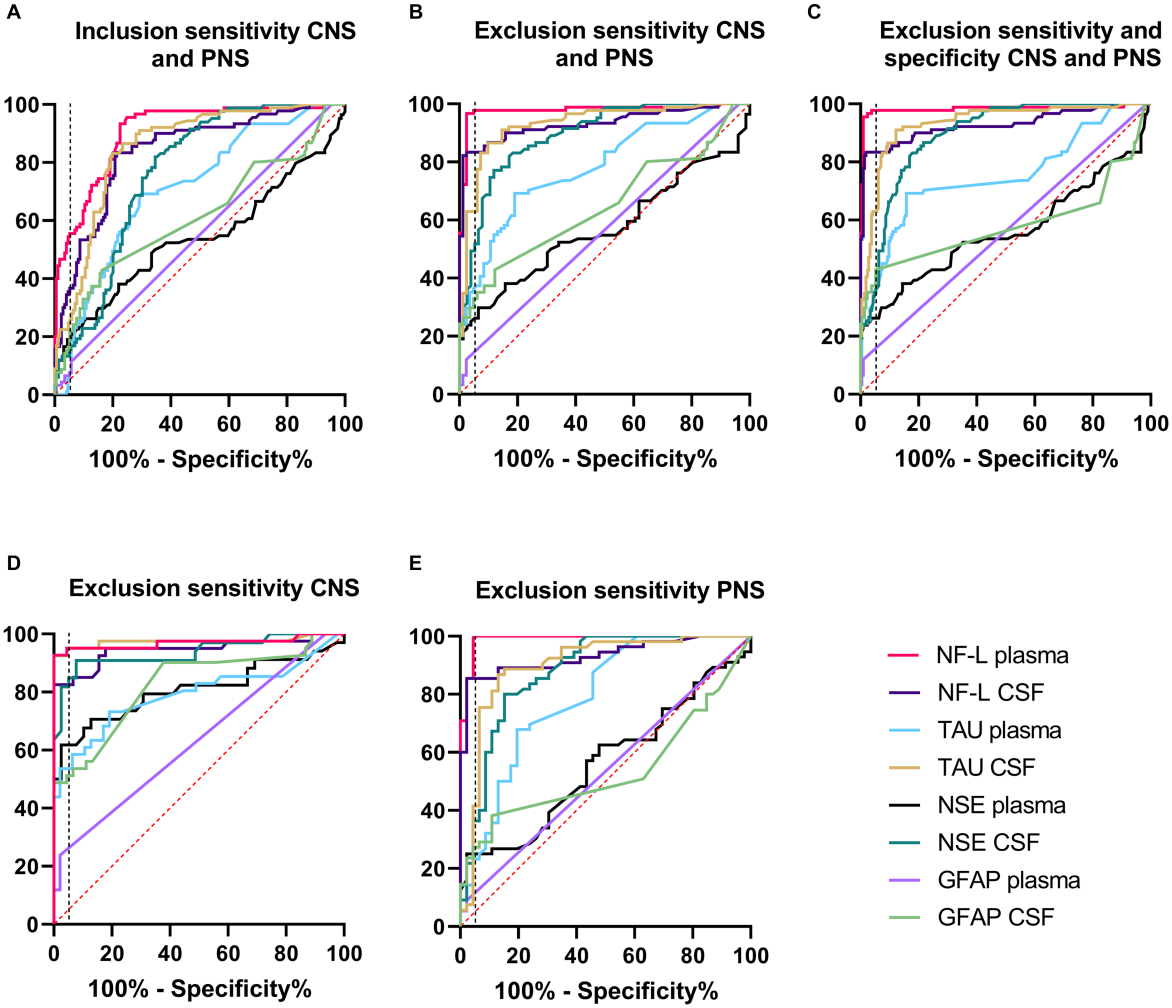
Receiver operating characteristics (ROC) curve analyses. Description and results for each analysis are summarized in Table 2. Red dash line represents non-discriminatory (random) result, the black dash line is set at 95% specificity. **(A)** Inclusion sensitivity CNS and PNS. **(B)** Exclusion sensitivity CNS and PNS. **(C)** Exclusion sensitivity and specificity CNS and PNS. **(D)** Exclusion specificity CNS. **(E)** Exclusion specificity PNS.

Receiver operating characteristics (ROC) curve analyses were conducted to allow for performance comparison between individual biomarkers. Results of ROC curves and analyses with the AUC and % sensitivity at 95% specificity are summarized in Table 2 and illustrated in Figures 2A–E. Table 2 was expanded to include confidence intervals for AUC and sensitivity values and is provided in Supplementary Table S7. To gain more detailed insight into the biomarker specificity and performance relative to the origin of the injury, five separate analyses were performed. The first analysis (letter A in the Table 2; Figure 2A), an inclusion sensitivity studies model, included all measured samples from sensitivity studies separated into categories: 1, no histopathological finding (controls and treated with histo score 0) and 2, treated with histopathological finding grade 1–5 in either CNS or PNS. In this analysis NF-L in plasma performed the best (AUC 0.90, sensitivity 53%). In the second analysis (letter B), an exclusion models with sensitivity studies, (Table 2B; Figure 2B), animals that were treated with NS toxicants, but with no test article-related histopathological findings in NS were excluded. This analysis avoids underestimation of biomarker sensitivities where their sensitivity is better than microscopic evaluation. Since there were numerous examples of biomarker increases without observable histopathologic changes in our studies, removal of those samples greatly improves the biomarker performance, especially in the sensitivity measure. In this analysis, NF-L is the best performer both in plasma and CSF with AUC and sensitivity 0.98; 97% and 0.93; 83%, respectively. Tau also performs well in CSF (AUC 0.93, sensitivity 63%). The third analysis (letter C) is an exclusion model with sensitivity and specificity studies (treated with non-NS toxicants) (Table 2C; Figure 2C). This analysis adds specificity studies to gain better insight into the specificity of the biomarkers. The overall parameters do not change much, indicating that biomarkers are not increased with other organ injuries and therefore are specific to the NS injury. The last two analyses were performed to gain better insight into the ability of the biomarkers to distinguish between CNS and PNS injury. They were an exclusion models consisting only of studies with either CNS or PNS toxicants. In the CNS model, performed as a fourth analysis (D), (Table 2D; Figure 2D), NF-L in plasma and Tau in CSF are the best performers with AUC 0.97 for both. NF-L and NSE in CSF also correlate well with the CNS injury well with AUC 0.94 and sensitivity of 82% for NF-L and 81% for NSE. In the final, fifth, analysis (E), an exclusion model with PNS studies only (Table 2E; Figure 2E), NF-L in plasma reaches AUC of 0.99 and 100% sensitivity followed by NF-L in CSF with AUC 0.93 and 85% specificity. Remaining biomarkers have low sensitivity measures.

**Table 2:** ROC curve analyses for candidate biomarkers.

		NF-L plasma	NSE plasma	Tau plasma	GFAP plasma	NF-L CSF	NSE CSF	Tau CSF	GFAP CSF
A. Inclusion sensitivity CNS and PNS (96 ctrl, 136 treated 0 histo and 84 treated with histo)	AUC	0.90	0.54	0.75	0.55	0.84	0.73	0.85	0.62
	Sensitivity %	53	17	3.3	6.5	17	13	23	16
	Cut-off	32	1.8	17	2.3	155	31	22	7.5
B. Exclusion sensitivity CNS and PNS (96 ctrl, and 84 treated with histo)	AUC	0.98	0.57	0.78	0.56	0.93	0.59	0.93	0.65
	Sensitivity %	97	26	36	10	83	49	63	31
	Cut-off	2.2	1.4	2.1	1.2	2.0	1.9	3.7	1.8
C. Exclusion sensitivity and specificity CNS and PNS (96 ctrl, 132 spec and 84 treated with histo)	AUC	0.98	0.56	0.75	0.56	0.93	0.89	0.93	0.58
	Sensitivity %	98	26	35	12	83	34	63	37
	Cut-off	2.1	1.4	2.2	1.1	2.0	2.5	3.8	1.1
D. Exclusion CNS (56 ctrl and 40 treated with histo)	AUC	0.97	0.80	0.79	0.63	0.95	0.94	0.97	0.83
	Sensitivity %	94	61	54	20	82	81	93	50
	Cut-off	2.2	1.4	1.9	1.3	1.7	1.7	3.2	2.5
E. Exclusion PNS (47 ctrl and 56 treated with histo)	AUC	0.99	0.56	0.79	0.53	0.93	0.88	0.90	0.53
	Sensitivity %	100	25	23	8	85	36	42	27
	Cut-off	2.8	0.6	2.2	1.1	2.7	2	3.8	1.4

### 3.3 Biomarker performance across all studies

Individual biomarker changes are captured in Figures 1A,B where biomarker changes were transformed to fold changes from the average of each study control group and group means and 95% CI were calculated. Studies in the plot were graphed as all study controls combined and then as study day necropsy groups and/or dose-groups, where applicable. Overall performance compared to the histopathological findings was summarized in Table 3. NF-L in plasma was the best-performing biomarker across all ROC analyses correctly identifying 97% of samples with NS toxicity and 100% samples with PNS toxicity. NF-L performance in CSF was slightly lower, but still very high with an AUC from 0.84 to 0.95. NF-L increased in a time- and/or dose-dependent manner and with increasing severity of nerve degeneration (seen in the NPA and acrylamide studies), demonstrating that NF-L CSF and plasma levels are proportionate to the extent of DINI. In several data subsets, NF-L measured in plasma and/or CSF demonstrated potential earlier detections of NS injuries than standard histomorphologic assessment, in the brain as demonstrated in the high dose of hexanedione, SD2 of CPA, and mid and high dose of amphetamine studies, as well as for PNS (SD4 of the doxorubicin study).

**Table 3:** Summary biomarker performance in studies with NS toxicity.

Biomarker	CNS					NS		PNS	
Study	CPA	MK-801	KA	AMPH	TMT	HXND	NPA	ACR	DOX
NF-L plasma	+++	++	+	+++	++	+++	++	++	+++
NF-L CSF	+++	+	+	+++	++	+++	++	++	+++
Tau plasma	−	+	+	+++	++	−	+	−	−
Tau CSF	+++	++	++	+++	++	+++	+	++	+
NSE plasma	+	−	+	−	n/a	−	−	−	−
NSE CSF	+++	−	++	+++	n/a	+++	+	−	−
GFAP plasma	+	−	−	+++	−	−	+	−	−
GFAP CSF	++	−	+	+++	+	+	+	−	−

+++ more sensitive than histopathology in some time groups or dose levels; ++ correlates with histopathology in most samples; + correlates with histopathology only in some samples; − does not increase with injury; n/a, not measured.

Total Tau performed well in CSF for studies with CNS damage, slightly out-performing NF-L and NSE but generally not responding well in plasma, even in samples with histopathologically confirmed damage. Tau was not increased in plasma in studies with PNS damage, however slight increases were observed in CNS of acrylamide and doxorubicin studies. NSE was increased only in CSF of studies with CNS damage but did not respond to peripheral damage, and therefore its presence in CSF could be confirmation of damage to brain neurons. GFAP, a marker of astrocyte damage, was the least responsive biomarker, increased only in studies with the highest degree of neuronal damage in the brain, mostly in CSF and to a lesser degree in plasma. Detection of GFAP in CSF and plasma could also, similarly to NSE, point to the CNS origin of the injury.

Taken together, NF-L was the best performer and a specific biomarker of NS system neuronal/axonal damage that appears to provide sensitivity that in some cases exceeds the standard histopathological assessment and increases before the onset of behavioral changes. Tau, NSE and GFAP were more sensitive when measured in CSF and could provide additional information about the localization of CNS or PNS injury.

## 4 Discussion

Four candidate protein biomarkers of NS damage were evaluated for their performance in plasma/serum and CSF of 15 sensitivity and specificity rat *in vivo* studies. Their performance was compared to behavioral and histopathological outcomes. All evaluated biomarkers have been previously reported as biomarkers of NS injury in human, mostly in various neurological diseases or traumatic brain injury (TBI) (Isgro et al., 2015; Yuan and Nixon, 2021; Abdelhak et al., 2022; Holper et al., 2022). Reports on DINI in human are scarcer and often limited to PNS injury with chemotherapeutics (Meregalli et al., 2021; Huehnchen et al., 2022). NF-L, the most widely studied NS marker, has been recently made available as a blood test in CLIA laboratories (LabCorp, Quanterix, Siemens), suggesting its potential for broader integration into routine clinical laboratory practice and clinical trials. Recent insights into pre-analytical factors have strengthened the understanding that NF-L is a relatively robust biomarker (Coppens et al., 2023). It is well known that NF-L levels increase with age, which complicates the establishment of the reference range (Hviid et al., 2020; Simrén et al., 2022) and perhaps makes it more difficult to diagnose NS injury in older individuals. Since NF-L is not specific to any particular disease, but rather a general marker of neuroaxonal injury, it is important to follow-up with finding the underlying cause. Even though the regulatory factors governing its concentration in biofluids, whether it’s passive release from injured axons, increased expression and release, impaired clearance, or a combination thereof, remains to be fully understood, the NF-L is poised for more extensive adoption in clinical laboratories. Thus, despite having validated precise assays for NF-L measurement in CSF and blood, various analytical, pre-analytical, and post-analytical aspects, including biomarker interpretation, must be considered throughout the entire NF-L testing process.

Evaluation in animals provides a unique opportunity to examine the biomarker responses to treatment and the correlation to the histopathologic outcome and behavioral changes in more detail. Reports of NS biomarkers in rats are not numerous and are mostly focused on NF-L. NF-L was reported to be increased in a rat study with vincristine-induced peripheral neuropathy (Meregalli et al., 2018), in serum of rats treated with trimethyltin, kainic acid and in pyridoxine-induced chromatolysis of the DRG (Sano et al., 2021), in cisplatin and paclitaxel-induced models of peripheral toxicity (Meregalli et al., 2020) and in monkey and rat with adeno-associated virus induced DRG toxicity (Fader et al., 2022). Similar to what was demonstrated in humans, NF-L has been reported to be released in serum in a rat model of TBI (O’Brien et al., 2021). GFAP and UCHL-1 were reported to be increased in rat CSF following kainic acid treatment (Glushakova et al., 2012).

Many of our studies incorporated several doses and/or time-points and we observed biomarker increases (NF-L plasma/CSF and Tau CSF) in groups where histopathological assessment by routine methods did not detect injury, yet later time-points and/or higher doses resulted in detectable DINI. In some cases, the NF-L-positive – histopathology-negative animals presented with behavioral changes in those groups (e.g., high dose of hexanedione and amphetamine) indicating presence of neurological effects that would potentially lead to observable histopathologic change as documented in the literature (Spencer and Schaumburg, 1975; Carvalho et al., 2012). In other cases (SD2 of CPA study and SD8 of doxorubicin study), there were no microscopic or behavioral changes, but the histopathological changes were present at a subsequent time-point. Detection of potential injuries in CNS or PNS could be challenging by a traditional histomorphological assessment using a selected representative portion or segment of brain or peripheral nerves for H&E stained sections, especially when the injury is localized. The current understanding of these NS biomarker candidates is that there are leakage biomarkers released into blood when cellular membrane integrity is lost (Yuan and Nixon, 2021) and thus are not predictive of future injury. Rather, they (NF-L in particular) appear to be extremely sensitive diagnostic tools for identifying degeneration or necrosis not readily detected by microscopic evaluation on limited tissue samples.

The assessment of the NS biomarkers in the large number of studies presented here (both in blood and CSF) provides several insights into each biomarker as well as several unexpected findings. For example, the ratio of NF-L concentration in CSF versus plasma observed here is somewhat variable on an individual animal basis, but approximately 1:40 on average, similar to what has been reported in human (Yuan and Nixon, 2021). It could be hypothesized that the NF-L CSF/plasma ratio would increase for injury originating in brain and decrease below the control ratio in studies with PNS injury. This theory holds true for a few studies with severe toxicity: the NF-L CSF/plasma ratio is on average about 1:200 for both days in the CPA study (CNS toxicity), but decreases to approximately 1:20 in the acrylamide and doxorubicin studies (PNS toxicity). This is not true, however, for the studies with smaller NF-L changes. MK-801 causes well documented CNS injury, localized to the posterior cingulate/retrosplenial cortex of the postero-medial aspect of the cerebrum of the brain (Fix et al., 2000), yet the NF-L increases in CSF are smaller than in plasma; this observation was repeated in an independent study with the same dose and with a necropsy at SD3 (data not shown).

Another, perhaps unexpected finding, was the presence of large amounts (up to 400-fold increase) of NF-L in the CSF collected in the studies with peripheral toxicity. In the doxorubicin study, neuron degeneration was detected in both peripheral nerve and DRG, but not in the brain. One explanation for the NF-L increases in CSF could be physical connections between the DRG and CSF. This hypothesis was also discussed by Sano in the pyridoxine model of peripheral injury with chromatolysis in the DRG where NF-L increases in plasma and CSF were reported (Sano et al., 2021). Direct communication of the rat spinal subarachnoid space and DRG was demonstrated previously (Joukal et al., 2016). An additional contributing factor for the presence of NF-L in CSF after doxorubicin treatment could be brain injury undetected by histopathological assessment. This hypothesis could be supported by observed minimal increases in Tau (4-fold) in CSF without any Tau increases in plasma. Similarly, in the acrylamide study, NF-L was increased in plasma up to 200-fold and in CSF up to 100-fold CSF with no histopathological damage to DRG, spinal cord or brain, yet with small but significant (T-test<0.001 for all groups) increases in Tau in CSF. Although CNS injury was not observed in the acrylamide study, acrylamide has been reported to induce central-peripheral distal axonopathy (Yu et al., 2005), and thus brain injury may have gone undetected.

Biomarker performance assessments are only as good as the analytical assays used for their measurement. None of the assays used here were developed specifically for rat; NF-L, NSE and GFAP are human assays and Tau is a mouse assay. Prior to our evaluations, the assays were evaluated for cross-reactivity and have shown to sensitively detect the four biomarkers in rat brain homogenates. However, when assayed in plasma, at a 1:2 dilution into the assay buffer, GFAP was below the level of detection in all control plasma samples and Tau in most of the measurements. Similarly, GFAP at 1:10 dilution in CSF was below the level of detection. Even though GFAP is expressed in the astrocytes (CNS) and non-myelinating Schwann cells (PNS) and therefore it is not a direct marker of neuronal damage as the other candidates, the lack of GFAP response is somewhat surprising, particularly in the context of publications on the successful use of GFAP as a biomarker in human (Yang and Wang, 2015). Although glial cell damage was not noted in any study by the histomorphologic assessments, it can be suggested that with extensive neuronal damage, glial cell damage occurs as well, with the expectation of this being monitorable by GFAP increases. With that in mind, one conclusion is that the assay itself is not optimal/sensitive enough for measuring rat GFAP. Similarly, even though Tau is present in peripheral axons, there were no increases noted with peripheral injury in our samples. Both in human and rats, Tau is expressed in several isoforms with a distinct, longer isoform (Big Tau) expressed in the periphery (Fischer and Baas, 2020; Holper et al., 2022). It is possible that the antibodies used in MSDIS Total Tau assay as described in the methods are not sensitive enough to detect the Big Tau in the rat. Even though we did not observed unexpectedly high NSE values in control samples, NSE could be increased in hemolyzed samples, since it is expressed in red blood cells, platelets, and lymphocytes (Motoyoshi et al., 2023).

Attention must also be paid to unexpected high values in CSF that can be caused by accidental introduction of nervous tissue during the CSF sampling. All biomarkers are present in large amounts in brain tissue, so that a very small amount of contamination can artificially increase levels of the biomarkers. Often, in such cases, all four biomarkers are measured at high levels, with Tau and NSE being especially sensitive to the smallest contamination from brain tissue in the CSF samples. A total of 19 CSF samples (some in controls and some from the treatment groups of the specificity studies) were eliminated from our sample set of approximately 450 samples for suspected contaminations.

Evaluation of the biomarkers in nonclinical settings provides many advantages in the assessment of performance. These advantages include the ability to evaluate different dose levels, timing of blood/CSF collections, and most importantly, a direct link to the histopathological outcomes to correlate with biomarker changes. Nonclinical evaluations further provide the groundwork for translation of the biomarkers from nonclinical drug development to the clinic with a more comprehensive understanding of the biomarker claims including sensitivity, specificity, and tissue/cell origin. Some of the studies presented here were conducted as part of two IMI projects, the NeuroDeRisk and TransBioLine, aiming to provide a better understanding of emerging biomarkers, both to help in de-risking drug candidates earlier in the research and development phases and to evaluate and qualify novel safety biomarkers including NF-L, Tau and GFAP, to monitor drug-induced NS injury for use in early clinical trials to ensure patient safety.

In summary, the performance of four NS biomarkers was evaluated in blood and CSF samples from 15 rat *in vivo* studies with CNS, PNS and non-NS organ injuries. NF-L was the overall best performing biomarker with sensitivity in detecting injury in both CNS and PNS. Additional biomarkers, like Tau in CSF, could provide added value by providing insight into the differentiation of CNS or PNS injuries.

The work presented here provides a compelling data set and lays a strong foundation for the further development and translation of these biomarkers for use in clinical settings, enabling studies towards continued validation of utility, qualification for use in clinical trials to assess drug safety, and implementation in clinical settings to further ensure patient safety.

## Data availability statement

The original contributions presented in the study are included in the article/Supplementary material, further inquiries can be directed to the corresponding author.

## Ethics statement

The studies were approved by the Merck & Co., Inc., Rahway, NJ, United States (MSD) or Pfizer Institutional Animal Care and Use Committee and conducted in an Association for Assessment and Accreditation of Laboratory Animal Care International–accredited facility in compliance with the National Institutes of Health (NIH) Guide for the Care and Use of Laboratory Animals. The studies were conducted in accordance with the local legislation and institutional requirements.

## Author contributions

KV: Data curation, Formal analysis, Writing – original draft. TT: Methodology, Writing – review & editing. IG: Methodology, Writing – review & editing. MR: Project administration, Writing – review & editing. CT: Data curation, Writing – review & editing. TD: Methodology, Writing – review & editing. CS: Project administration, Writing – review & editing. LC: Project administration, Writing – review & editing. WG: Supervision, Writing – review & editing.
