# Supplementary material for: Performance of biomarkers NF-L, NSE, Tau and GFAP in blood and cerebrospinal fluid in rat for the detection of nervous system injury
Source: Front Neurosci. 2024 Jan 16;17:1285359. doi: 10.3389/fnins.2023.1285359 (PMC10824906; doi:10.3389/fnins.2023.1285359)
Supplement: Supplementary file 1 [file Table_1.DOCX]

Supplementary Material

Performance of Biomarkers NF-L, NSE, Tau and GFAP in blood and Cerebrospinal Fluid in Rat for the Detection of Nervous System Injury

**Katerina Vlasakova^1^*, Takayuki Tsuchiya^1^, Ivy N Garfinkel^1^, Michael P Ruth^1^, Cheryl Tyszkiewicz^2^, Theodore J Detwiler^1^, Christopher J Somps^3^, Lorenzo Di Cesare Mannelli^4^, Warren E. Glaab^1^**

*** Correspondence:** Katerina Vlasakova

Katerina_vlasakova@merck.com

# Supplementary Tables

**Supplementary Table 1**

Functional Observation Battery Assessment in the 2-Chloropropionic acid Study 24 and 48 Hours Post-dose

| FOB Parameter | Observations | 24 Hours Postdose (n=10) | 48 Hours Postdose (n=9)^a^ |
| --- | --- | --- | --- |
| Activity, Posture & Gait | Decreased activity in Home Cage | - | 8 |
|  | Increased activity in Home Cage | - | 1 |
|  | Decreased activity in Open Field: 0-2 lines (during 2-min obs) | - | 5 |
|  | Decreased activity in Open Field: 0-2 rears (during 2-min obs) | 1 | 9 |
|  | No hindlimb grip strength (would not grip bars OR too impaired) | 1 | 2 |
|  | No foot splay (animal too impaired for test) | - | 9 |
|  | Flat posture | 1 | 2 |
|  | Hunched posture | - | 3 |
|  | Forelimbs extended | - | 3 |
|  | Hindlimbs splayed | - | 6 |
|  | Sternally recumbent (in home cage &/or open field) | 1 | 7 |
|  | Intermittent curled forelimbs | 1 | - |
|  | Dragging body with forelimbs / hindlimbs or stomach dragging | 1 | 3 |
|  | Swaying side-to-side / Unsteady Gait / Ataxia | - | 6 |
|  | Leaning &/or circling (left or right) | - | 3 |
|  | Difficult to remove from cage | - | 1 |
| Reflexes | Slow surface righting | 2 | 5 |
|  | Impaired surface righting | - | 4 |
|  | Slow aerial righting | 2 | ^b^ |
|  | No response to approach until whiskers touched | - | 1 |
|  | Energetic click response | 2 | 2 |
|  | No touch response | - | 2 |
|  | Energetic tail pinch response | 1 | 1 |
|  | No tail pinch response | 1 | 4 |
| Uncontrolled / Repetitive Movements | Tail - wagging, straub (rigid) | - | 3 |
|  | Head jerking or moving side-to-side | - | 2 |
|  | Ears twitching | - | 2 |
|  | Teeth chattering | - | 1 |
|  | Tonic convulsion (i.e. body rigidity, falling over, eyes closed) | - | 4 |
|  | Clonic-tonic convulsions (clonic includes: forelimb paddling and/or alternating contraction/relaxation of muscles) | - | 2 |
| Physical Signs | Slight, red lacrimation | - | 2 |
|  | Red discharge from nose | - | 2 |
|  | Slight salivation | - | 1 |
|  | Decreased muscle tone | - | 1 |
|  | Decreased body temperature (compared to controls) | 2 | 8 |
| FOB Measurements as Compared to the Control Group Mean | | | |
| Mean Measurements | Decreased lines | - | -92% |
|  | Decreased rears | - | -96% |
|  | Decreased body temperature | - | -1.3°C |

^a^ One animal euthanized prior to FOB assessment due to clinical signs. ^b^ Measurement not conducted on any animals due to impairment.

- = No test article-related change

**Supplementary Table 2A**

Functional Observation Battery (FOB) Assessment in the Acrylamide Acid Study at 40 mg/kg/dose at Study Week 2, 3 and 5

| FOB Findings^a^ | | Study Week 2 (n=10) | Study Week 3 (n=6) | Study Week 5 (n=6) |
| --- | --- | --- | --- | --- |
| Gait & Muscle Tone | Ataxia – hindlimbs | 4 | 1 | 6 |
|  | Walking slowly | - | 1 | - |
|  | Decreased muscle tone | - | 3 | 6 |
| Stimulus Activity Response | Impaired aerial righting reflex | - | - | 4 |
| Uncontrolled and/or Repetitive Movements | Intermittent whole-body trembling during defecation | - | - | 1 |
| Behavior | Labored breathing during defecation | - | - | 2 |
| General Appearance | Distended abdomen | - | 1 | 1 |
| FOB Measurements as Compared to the Control Group Mean | | | | |
| # of Line Crosses | Decreased | - | -46% | -50% |
| # of Rears | Decreased | - | -50% | -57% |
| Forelimb Grip Strength | Decreased | - | -13% ^b^ | - |

^a^ Data presented as the incidence of the findings within each group. ^b^ Observed in two animals only. - = No test article-related change

**Supplementary Table 2B**

Functional Observation Battery Assessment in the Acrylamide Acid Study at 60 mg/kg/dose at Study Week 2, 3 and 5

| FOB Findings^a^ | | Study Week 2 (n=10) | Study Week 3 (n=6) | Study Week 5 (n=6) |
| --- | --- | --- | --- | --- |
| Gait & Muscle Tone | Ataxia - hindlimbs | 5 | 6 | - |
|  | Walking on hind toes | 1 | - | - |
|  | Hindlimbs slipping during walk | - | 1 | - |
|  | Hindlimbs slipping/sliding during walk | - | 2 | - |
|  | Splayed/limited use/dragging hindlimbs | - | 2 | 6 |
|  | Curling forelimb(s)/paw(s) | - | 1 | 2 |
|  | Decreased muscle tone | - | 3 | 6 |
|  | Sternal recumbency | - | - | 6b |
| Stimulus Activity Response | Impaired surface righting reflex | - | - | 5 |
|  | Impaired aerial righting reflex | - | - | 3 |
|  | Aerial righting not conducted due to impairment | - | - | 3 |
| Behavior | Decreased activity in home cage | - | - | 4 |
| General Appearance | Audible respiratory sounds | - | - | 1 |
|  | Distended abdomen | - | - | 5 |
|  | Fecal staining | - | - | 1 |
| FOB Measurements as Compared to the Control Group Mean | | | | |
| # of Line Crosses | Decreased | - | -46% | -68% |
| # of Rears | Decreased | - | -50% | -100% |
| Forelimb Grip Strength | Decreased | - | -15% | -47% |
| Hindlimb Foot Splay | Increased | - | +33% | c |
| Body Temperature | Decreased | - | -0.7°C | -0.4°C |

^a^ Data presented as the incidence of the findings within each group. ^b^ Intermittent for 2 animals. ^c^ N = 1 (not conducted on remaining animals due to impairment). - = No test article-related change

**Supplementary Table 3**

Functional Observation Battery Assessment in the Doxorubicin Study at Study Day 4, 7 and 14

| FOB Parameter | | Observation | 4 days postdose (n=8) | 7 days postdose (n=8) | 14 days postdose (n=8) |
| --- | --- | --- | --- | --- | --- |
| Activity, Posture & Gait | | Decreased line crosses (compared to controls) | 2 | 7 | 5 |
|  |  | Decreased rears (compared to controls) | 3 | 4 | 2 |
|  |  | Decreased hindlimb grip strength (compared to controls) | 5 | 2 | 1 |
|  |  | Flattened posture | - | 2 | 1 |
|  |  | Hunched posture | 1 | - | 1 |
|  |  | Decreased muscle tone | 1^a^ | - | 6 |
| Reflexes | | Slow surface righting | 1 | - | - |
| Additional Physical Signs | | Unformed feces | 1 | - | - |
|  |  | Vocal upon handling | - | 1 | - |
|  |  | Piloerection | - | - | 4 |
|  |  | Alopecia - under jaw and/or ventral cervical | - | - | 2 |
|  |  | Slight palpebral closure / Eyes partially closed - intermittent | - | - | 2 |
|  |  | Lacrimation - dried, black | - | - | 3 |
| Body Temperature | | Decreased body temperature | 5 | 8 | 4 |
| FOB Measurements as Compared to the Control Group Mean | | | | | |
| Mean Measurements | | Decreased line crosses | - | -52% | -39% |
|  |  | Decreased rears | - | -43% | -43% |
|  |  | Hindlimb grip strength | - | - | - |
|  | Decreased body temperature | | -0.3°C | -0.7**°**C | -0.3°C |

a = animal also had unformed feces. - = not observed or insignificant mean change from control mean

**Supplementary Table 4**

Functional Observation Battery Assessment in the Kainic Acid Study After Dosing and 24 hours Post-dose

| Incidence of Treatment-Related Findings (n=12) | | | |
| --- | --- | --- | --- |
| Study Day 1 (FOB Home Cage Only) | Activity prior to onset of most findings | Active/alert/walking at ~0.75-1 hour postdose  (as compared to the controls which had 5/6 sleeping at this time) | 12 |
|  | Uncontrolled and/or Repetitive Movements (stereotypy) | Full body shakes (wet dog shakes) | 11 |
|  |  | Head trembling, twitching or jerking | 4 |
|  |  | Head bobbing | 1 |
|  |  | Chewing | 4 |
|  |  | Forelimb twitching | 1 |
|  |  | Full body twitching | 2 |
|  |  | Full body rigidity and straub tail | 1a |
|  |  | Rapid head movements and rapid pawing; retropulsion (reverse walking); rapid rearing and falling over | 1a |
|  |  | Rearing with forelimb paddling | 1 |
|  |  | Increased activity w/ rapid head movements | 1 |
|  | Physical Appearance | Mouth - salivation | 1 |
|  |  | Mouth - salivation with red tinge | 1 |
|  |  | Mouth - foam | 3 |
|  |  | Nose - red discharge | 1 |
|  |  | Left or both eyes - partially closed | 3 |
|  | Early Termination | Early Sacrifice (~2.75 hours postdose) | 1a |
| Incidence of Treatment-Related Findings (n=11) | | | |
| Study Day 2 (routine FOB) | Behavior | Difficult to remove from cage | 3 |
|  |  | Aggressive | 1 |
|  |  | Increased muscle tone | 1 |
|  | Activity | Decreased number of rears (at least -40% less than lowest control) | 2 |
|  | Physical Appearance | Muzzle - red discoloration | 1 |
|  |  | Ears - reddened | 3 |
|  |  | Piloerection; hunched; red discoloration: muzzle, abdomen, tail, all limbs and forepaws; red urine | 1 |
|  | Stimulus Activity Response | Dilated pupils | 1 |
|  |  | Constricted pupils | 5 |

^a^ Due to the severity and duration of signs related to clonic-tonic convulsion, this animal was sent to early necropsy prior to the end of the 3 hour FOB home cage observations

**Supplementary Table 5**

Functional Observation Battery Assessment in the MK-801 Study After Dosing and 24 hours Post-dose

| Incidence of Treatment-Related Findings (n=12) | | | |
| --- | --- | --- | --- |
| Study Day 1 (FOB Home Cage Only) | Activity and/or Gait | Circling (onset = ~3-4 min PD) | 6 |
|  |  | Falling side to side (onset = ~3-4 min PD) | 12 |
|  |  | Recumbent (onset = ~7-30 min PD) | 12 |
|  |  | Sternal recumbent & motionless w/ eyes wide open (onset = ~1.5 to 2.5 hours postdose) | 8 |
|  | Uncontrolled and/or Repetitive Movements (stereotypy) | Head - bobbing, weaving | 4 |
|  |  | Head - trembling or twitching | 3 |
|  |  | Chewing | 1 |
|  |  | Hindlimb twitching | 1 |
|  |  | Hindlimb paddling | 3 |
|  |  | All limbs paddling | 1 |
|  |  | Full body - trembling or twitching | 6 |
|  |  | Full body - rigidity | 2 |
|  | Physical Appearance | Eyes wide open (prior to being recumbent) | 8 |
|  |  | Straub tail | 5 |
|  |  | Mouth - salivation | 3 |
|  |  | Eyes - red discharge | 1 |
| SD 2 | Early Termination | Early sacrifice on Study Day 2 | 12^a^ |

^a^ Due to continuation and severity of signs observed on Study Day 2, all animals were sent to early necropsy on Study Day 2. SD = Study Day

**Supplementary Table 6**

Functional Observation Battery Assessment in the 3-NPA Study at Study Day 7 and 14.

| FOB Findings | | 12.5 mg/kg b.i.d.  Study Day 7 | 7.5 mg/kg b.i.d.  Study Day 7 | 7.5 mg/kg b.i.d.  Study Day 14 |
| --- | --- | --- | --- | --- |
| Physical Appearance | Decreased home cage activity | 1 | - | 1 |
|  | Decreased muscle tone | 5 | 1 | 2 |
|  | Piloerection | 1 | - | - |
|  | Tail - curled upwards | - | - | 1 |
|  | Vocalization upon handling | 1 | - | - |
| Posture | Hunched posture | 4 | - | 1 |
|  | Sternally recumbent | 2 | - | - |
|  | Leaning left and/or right | - | - | 2 |
| Gait | Ataxia | 4 | 1 | 1 |
|  | Hindlimb(s) – dragging, dangling or limited use | 3 | 2 | 2 |
|  | Hindlimb(s) – no use | 1 | - | - |
|  | Hindfeet or toes - curled | 2 | 2 | - |
|  | Hindlimbs - intermittent hopping | - | - | 1 |
|  | Hindlimbs - splayed | 2 | - | - |
| Uncontrolled and/or Repetitive Movements (stereotypy) | Turning head side to side | 1 | - | - |
| Stimulus Activity Response | Pupils dilated (didn’t constrict in response to light) | 2 | - | - |
|  | No approach response | 1 | - | - |
|  | No pinna response | 1 | - | - |
|  | No tail pinch (nociception) response | 2 | - | - |
|  | Slow or impaired surface righting | 4 | - | 1 |
|  | Slow or impaired aerial righting | 1 | - | - |
|  | Aerial righting not taken^a^ | 4 | 1 | 1 |
| Foot Splay | Not taken^a^ | 4 | 1 | 2 |

| FOB Measurements as Compared to the Control Group Mean^b^ | | | | |
| --- | --- | --- | --- | --- |
| Locomotor Activity | Fewer # of lines crossed | 3 (-80%) | 1c | 1c |
|  | Fewer (or no) rears | 4 (-88%) | - | 3 (-40%) |
| Body Temperature | Lower | 5 (-1.5ºC) | - | 1 c |
| Hindlimb Grip | Lower grip strength | 1[1]^c^ | 1[4]^c^ | 1[4]^c^ |
|  | Not taken^a^ | 4 | 1 | 1 |

N = 5 for all parameters, except for lower hindlimb grip strength – the number in [ ] is the number of animals evaluated. - = No change. Not taken due to impairment. ^b^ Number of individual findings with group change or % change (compared to the control group mean) in parenthesis, where applicable. ^c^ Due to finding limited to only a few animals within a dose group, group mean data compared to the control does not apply. b.i.d = twice daily

**Supplementary Table 7**

Receiver Operating Characteristics (ROC) Curve Analyses for Candidate Biomarkers with added Confidence Interval (CI)

|  |  | NF-L plasma | NSE plasma | Tau plasma | GFAP plasma | NF-L  CSF | NSE CSF | Tau CSF | GFAP CSF |
| --- | --- | --- | --- | --- | --- | --- | --- | --- | --- |
| A. Inclusion sensitivity CNS and PNS (96 ctrl, 136 treated 0 histo & 84 treated with histo | AUC  AUC CI | 0.90  0.87-0.94 | 0.54  0.46-0.62 | 0.75  0.65-0.78 | 0.55  0.48-0.62 | 0.84  0.79-0.89 | 0.73  0.70-0.82 | 0.85  0.81-0.90 | 0.62  0.54-0.69 |
|  | Sensitivity %  Sensitivity % CI | 53  43-63 | 17  14-27 | 3.3  0.9-9 | 6.5  3-14 | 17  10-27 | 13  8-22 | 23  16-33 | 16  9-25 |
|  | cut-off | 32 | 1.8 | 17 | 2.3 | 155 | 31 | 22 | 7.5 |
| B. Exclusion sensitivity CNS and PNS (96 ctrl, & 84 treated with histo) | AUC  AUC CI | 0.98  0.95-1 | 0.57  0.48-0.69 | 0.78  0.71-0.85 | 0.56  0.48-0.65 | 0.93  0.89-0.97 | 0.59  0.85-0.94 | 0.93  0.89-0.97 | 0.65  0.57-0.73 |
|  | Sensitivity %  Sensitivity % CI | 97  91-99 | 26  17-36 | 36  26-47 | 10  3-20 | 83  74-90 | 49  39-60 | 63  53-72 | 31  21-43 |
|  | cut-off | 2.2 | 1.4 | 2.1 | 1.2 | 2.0 | 1.9 | 3.7 | 1.8 |
| C. Exclusion sensitivity and specificity CNS and PNS (96 ctrl, 132 spec & 84 treated with histo | AUC  AUC CI | 0.98  0.94-1 | 0.56  0.48-0.65 | 0.75  0.69-0.82 | 0.56  0.49-0.63 | 0.93  0.89-0.97 | 0.89  0.86-0.93 | 0.93  0.90-0.97 | 0.58  0.50-0.67 |
|  | Sensitivity %  Sensitivity % CI | 98  92-100 | 26  18-36 | 35  24-45 | 12  3-20 | 83  74-90 | 34  24-46 | 63  53-72 | 37  28-47 |
|  | cut-off | 2.1 | 1.4 | 2.2 | 1.1 | 2.0 | 2.5 | 3.8 | 1.1 |
| D. Exclusion CNS (56 ctrl & 40 treated with histo | AUC  AUC CI | 0.97  0.93-1 | 0.80  0.70-0.92 | 0.79  0.69-0.89 | 0.63  0.52-0.75 | 0.95  0.89-1 | 0.94  0.88-1 | 0.97  0.93-1 | 0.83  0.74-0.92 |
|  | Sensitivity %  Sensitivity % CI | 94  81-99 | 61  45-76 | 54  39-66 | 20  13-39 | 82  68-91 | 81  66-91 | 93  80-99 | 50  34-66 |
|  | cut-off | 2.2 | 1.4 | 1.9 | 1.3 | 1.7 | 1.7 | 3.2 | 2.5 |
| E. Exclusion PNS (47 ctrl & 56 treated with histo | AUC  AUC CI | 0.99  0.97-1 | 0.56  0.45-0.67 | 0.79  0.7-0.88 | 0.53  0.42-0.65 | 0.93  0.88-0.98 | 0.88  0.8-0.95 | 0.90  0.83-0.97 | 0.53  0.42-0.65 |
|  | Sensitivity %  Sensitivity % CI | 100  93-100 | 25  16-38 | 23  14-36 | 8  4-19 | 85  74-92 | 36  25-50 | 42  29-55 | 27  17-40 |
|  | cut-off | 2.8 | 0.6 | 2.2 | 1.1 | 2.7 | 2 | 3.8 | 1.4 |

AUC = Area under the curve.
